# Success Rates of Dental Implants in Patients With Diabetes: A Systematic Review

**DOI:** 10.7759/cureus.76361

**Published:** 2024-12-25

**Authors:** Yashab James, Wajeeh Mohsin M Butt, Habiba Shahid, Shayzmin Ahmad, Muhammad Talha Bin Imran, Nouman Anthony

**Affiliations:** 1 Dentistry, Abbottabad Medical College, Peshawar, PAK; 2 Dentistry, Pakistan Institute of Medical Sciences, Islamabad, PAK; 3 General Medicine, Rehman Medical Institue, Peshawar, PAK

**Keywords:** clinical outcomes, dental implants, glycemic control, implant survival, marginal bone loss, peri-implant biomarkers, systematic review, type 2 diabetes mellitus

## Abstract

This systematic review evaluates the success rates of dental implants in patients with type 2 diabetes mellitus, focusing on outcomes such as implant survival, marginal bone loss, and peri-implant biomarkers. A comprehensive literature search was conducted across multiple databases, including PubMed, Cochrane, Embase, Scopus, and Web of Science, to identify relevant systematic reviews and meta-analyses. Four studies were included, encompassing diverse populations and interventions. Findings indicate that diabetes, when well-managed (HbA1c < 8%), does not significantly compromise implant survival rates, with survival percentages ranging from 96.1% to 97.3% at one year and 87.3% to 96.1% at five years, comparable to non-diabetic populations. However, peri-implant health metrics, such as marginal bone loss (mean difference: -0.08 mm; 95% CI: -0.25 to 0.08) and probing depth, were adversely affected in poorly controlled diabetes (HbA1c > 8%), highlighting the critical role of glycemic control. Advanced statistical approaches, including dose-response relationships, revealed a progressive worsening of peri-implant outcomes as HbA1c levels increased. The review underscores the importance of interdisciplinary care and strict adherence to clinical protocols to optimize outcomes for diabetic patients receiving dental implants. Despite robust findings, limitations include heterogeneity among included studies and the need for long-term data to validate the observed trends. Future research should focus on standardized reporting and exploring the impact of advanced glycemic thresholds on implant success.

## Introduction and background

Dental implants are a widely accepted and effective solution for replacing missing teeth, offering improved functionality, aesthetics, and patient satisfaction compared to traditional dentures or bridges [1]. Globally, the use of dental implants has grown significantly, with millions of procedures performed annually, and their adoption among patients with systemic conditions, including diabetes mellitus, is steadily increasing. However, the success of dental implants can be influenced by a variety of systemic conditions, including type 2 diabetes mellitus (T2DM), which is characterized by chronic hyperglycemia and associated metabolic disturbances [2]. Diabetes has been identified as a potential risk factor for implant failure due to its impact on bone metabolism, delayed wound healing, and an increased susceptibility to infections [3]. As the global prevalence of diabetes continues to rise - affecting approximately 10.5% of the adult population worldwide - understanding its implications on dental implant success is crucial for improving clinical outcomes and optimizing patient care.

T2DM, the most common form of diabetes, has a direct impact on the osseointegration process - a critical factor for the stability and longevity of dental implants [4]. Several studies have explored the survival rates and peri-implant outcomes in diabetic patients, but the findings remain inconsistent, with some indicating comparable success rates to non-diabetic individuals and others suggesting a significantly higher risk of complications [5]. To address this discrepancy, it is imperative to systematically evaluate the current evidence, focusing on survival rates, peri-implant health, and other clinical parameters in diabetic patients undergoing implant placement. This systematic review aims to synthesize the available literature to provide a comprehensive understanding of the success rates of dental implants in patients with diabetes, highlighting potential predictors of success and failure.

The Problem, Intervention, Comparison and Outcome (PICO) framework provides a structured approach to framing the research question, guiding the selection and evaluation of studies included in this systematic review [6]. The target population includes patients diagnosed with diabetes mellitus, specifically focusing on those with type 2 diabetes. Studies involving individuals with poorly controlled or well-managed glycemic levels will be considered to capture a wide spectrum of clinical scenarios. The intervention of interest is the placement of dental implants, including immediate and delayed loading protocols. The review will also account for variations in implant designs, materials, and surgical techniques to evaluate their influence on success rates in diabetic patients. The primary comparison will be between diabetic and non-diabetic individuals undergoing dental implant placement. Studies comparing outcomes within diabetic subgroups, such as those with varying levels of glycemic control, will also be included. The primary outcomes of interest include implant survival rates, peri-implant health (measured by bone loss, pocket depth, and bleeding on probing), and the incidence of complications such as implant failure and peri-implantitis. Secondary outcomes may include patient satisfaction and quality of life. By addressing the PICO elements, this systematic review seeks to answer the critical question: “What are the success rates of dental implants in patients with diabetes, and how do these rates compare to non-diabetic individuals or diabetic subgroups with varying levels of glycemic control?”

## Review

Materials and methods

Search Strategy

A comprehensive search strategy was employed to ensure the inclusion of all relevant studies addressing the success rates of dental implants in patients with diabetes. Multiple databases, including PubMed, Cochrane, Embase, Scopus, and Web of Science, were systematically searched using a combination of keywords and MeSH terms such as "dental implants", "diabetes mellitus", "glycemic control", "implant survival", and "marginal bone loss". The search was not restricted by date but was limited to peer-reviewed articles in English to maintain quality and accessibility. Additional sources were identified through manual searches of reference lists and gray literature to minimize publication bias. This review adhered to the Preferred Reporting Items for Systematic Reviews and Meta-Analyses (PRISMA) [7] guidelines, ensuring transparency and rigor in the study selection, screening, and data extraction processes. The structured search strategy allowed for a robust and inclusive analysis of the current evidence base.

Eligibility Criteria

The eligibility criteria for this systematic review were defined using the PICO framework to ensure a focused and comprehensive selection of studies. Included studies had to involve patients with diabetes mellitus, specifically type 2 diabetes, undergoing dental implant placement, with outcomes such as implant survival rates, marginal bone loss, and peri-implant biomarkers. Only systematic reviews and meta-analyses were included to provide a high level of evidence. Studies in English, peer-reviewed journals, and published up to the most recent database search date were considered. To differentiate between levels of diabetes control, studies were required to specify glycemic control status using objective markers, such as hemoglobin A1c (HbA1c) levels. Well-controlled diabetes was defined as HbA1c ≤ 7%, while poorly controlled diabetes was classified as HbA1c > 8%, based on established clinical thresholds. Exclusion criteria included studies focusing solely on non-diabetic populations, case reports, and reviews without meta-analytic components or data relevant to implant success.

Additional criteria required studies to report on peri-implant health outcomes, including probing depth and inflammation, with clear differentiation between diabetic and non-diabetic groups or subgroups based on glycemic control. Articles with ambiguous reporting, incomplete data, or populations with other uncontrolled systemic diseases were excluded to maintain consistency and relevance. This rigorous inclusion and exclusion process ensured that the review synthesized data directly pertinent to understanding the success of dental implants in diabetic populations.

Data Extraction

Data extraction was performed systematically to ensure consistency and accuracy. A standardized extraction form was used to collect key information from each included study, including author names, publication year, study design, sample size, population characteristics (e.g., diabetic versus non-diabetic groups, glycemic control levels), intervention details (e.g., type of implants, loading protocols), and reported outcomes such as implant survival rates, marginal bone loss, and peri-implant biomarkers. Statistical measures, including relative risks, mean differences, confidence intervals, and p-values, were also extracted to facilitate comparison and synthesis. Two independent reviewers conducted the data extraction process, with discrepancies resolved through discussion or consultation with a third reviewer to ensure reliability and minimize bias.

Data Analysis and Synthesis

The extracted data were synthesized qualitatively to provide a comprehensive understanding of the findings across the included studies. Key outcomes such as implant survival rates, marginal bone loss, and peri-implant health metrics were compared descriptively, emphasizing trends and patterns among diabetic and non-diabetic populations. Subgroup analyses were explored narratively, focusing on variations in outcomes based on glycemic control levels and other patient factors. Heterogeneity in study designs, populations, and reported outcomes was acknowledged and discussed to ensure transparency. The findings were integrated into a cohesive narrative, highlighting the implications for clinical practice and identifying areas for further research. This qualitative synthesis provided valuable insights without statistical pooling, offering a clear interpretation of the current evidence.

Results

Study Selection Process

**Figure 1:**
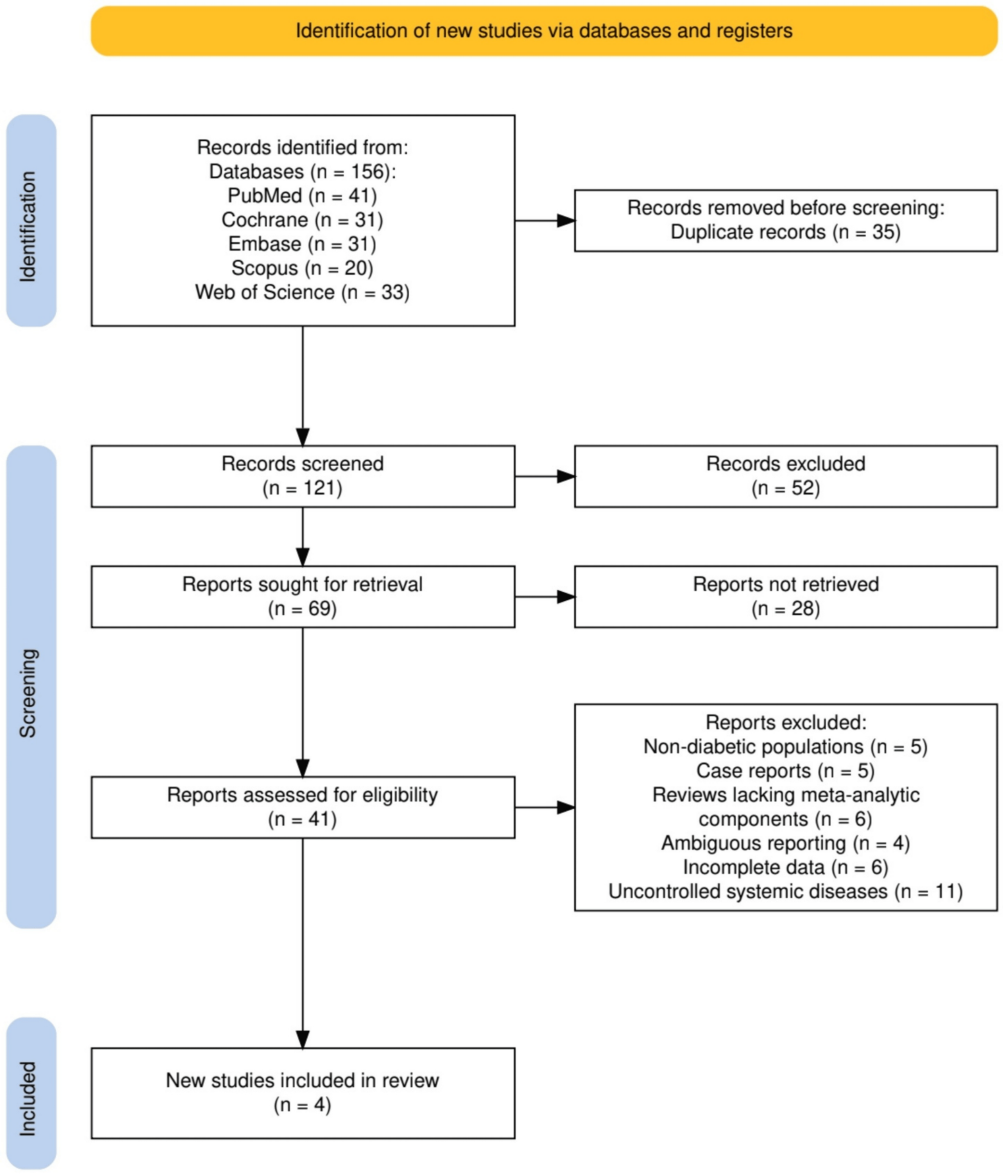
The PRISMA flowchart represents the study selection process. PRISMA: Preferred Reporting Items for Systematic Reviews and Meta-Analyses

The study selection process involved several rigorous steps to ensure the inclusion of relevant and high-quality studies, as depicted in Figure 1. A total of 156 records were identified through comprehensive database searches, including PubMed (41), Cochrane (31), Embase (31), Scopus (20), and Web of Science (33). After removing 35 duplicate records, 121 records underwent screening based on titles and abstracts, resulting in the exclusion of 52 studies for not meeting the inclusion criteria. Of the 69 reports sought for retrieval, 28 could not be accessed. Subsequently, 41 reports were assessed for eligibility, with 37 excluded due to factors such as non-diabetic populations (5), case reports (5), reviews lacking meta-analytic components (6), ambiguous reporting (4), incomplete data (6), and uncontrolled systemic diseases (11). Ultimately, four studies met all the inclusion criteria and were included in the final systematic review. This methodical selection process adhered to PRISMA guidelines to ensure transparency and rigor in synthesizing the evidence.

Characteristics of the Selected Studies

The selected studies, summarized in Table 1, encompass systematic reviews and meta-analyses investigating the success rates of dental implants in diabetic populations. The studies included diverse patient groups, ranging from geriatric patients with systemic conditions to individuals with T2DM undergoing various implant loading protocols. Key interventions analyzed included immediate and conventional loading of dental implants, with comparisons drawn between diabetic and non-diabetic populations. Outcomes measured spanned implant survival rates, marginal bone loss, and peri-implant biomarkers, with follow-up durations ranging up to six years. Statistical findings demonstrated consistently high implant survival rates, even in diabetic patients with controlled glycemic levels, although marginal bone loss and peri-implant health metrics were adversely impacted in cases of poor glycemic control. These studies provided valuable insights into the impact of diabetes on dental implant success, highlighting the importance of glycemic management and clinical protocol adherence.

**Table 1:** Summary of included studies on dental implant success in diabetic patients. AGEs: Advanced Glycation End Products; BOP%: Bleeding on Probing Percentage; CI: Confidence Interval; DM/HG: Diabetes Mellitus/Hyperglycemia; DRMA: Dose-Response Meta-Analysis; HbA1c: Hemoglobin A1c; IL: Immediately Loaded; IL-6: Interleukin-6; IL-8: Interleukin-8; ISQ: Implant Stability Quotient; MBL: Marginal Bone Loss; MD: Mean Difference; PD: Probing Depth; PICF: Peri-Implant Crevicular Fluid; PRISMA: Preferred Reporting Items for Systematic Reviews and Meta-Analyses; RANKL: Receptor Activator of Nuclear Factor Kappa-Β Ligand; RR: Relative Risk; TNF-α: Tumor Necrosis Factor Alpha

Author(s) and Year	Study Design	Population (Sample Size)	Intervention	Comparison	Outcome(s)	Statistics	Key Findings
Schimmel et al., 2018 [8]	Systematic Review and Meta-Analysis	Geriatric patients (≥75 years) and patients with systemic conditions (N = 60 studies, 7 for meta-analysis)	Dental implant placement	Healthy patients and patients with various conditions (e.g., diabetes, Parkinson’s, cancer)	Implant survival rates at 1 and 5 years	1-year survival: 97.3% (95% CI: 94.3–98.7); 5-year survival: 96.1% (95% CI: 87.3–98.9)	Implant survival was high (97.3% at 1 year, 96.1% at 5 years). Diabetes mellitus type II and Parkinson’s had high survival rates, but cancer patients experienced lower rates due to radiotherapy.
Andrade et al., 2022 [9]	Systematic Review and Meta-Analysis	Type 2 diabetic patients undergoing implant placement (7 studies; 5 for meta-analysis)	Immediately loaded (IL) dental implants	Non-diabetic patients and conventional loading in diabetic patients	Implant survival rates, peri-implant marginal bone loss	Survival rate: RR = 1.00 (95% CI: 0.96–1.04, p = 0.91); Marginal bone loss: MD = -0.08 (95% CI: -0.25–0.08, p = 0.33)	No significant difference in implant survival between diabetic and non-diabetic groups, even with poor glycemic control. Marginal bone loss between IL and conventional loading showed no significant difference. Strict protocol adherence and glycemic control are critical for success.
Lv et al., 2022 [10]	Systematic Review and Meta-Analysis	634 participants from 10 studies with follow-up up to 6 years	Dental implant placement in DM/HG patients	Healthy individuals	Peri-implant biomarkers, clinical and radiographic outcomes, implant survival	AGEs in PICF: p < 0.01; ISQ: p = 0.04; BOP%: p < 0.01; PD: p = 0.01; MBL: p < 0.01; DRMA: PD and MBL worsened with HbA1c > 8%	Negative regulators of bone metabolism (IL-6, TNF-α, IL-8, RANKL) were significantly higher in the DM/HG group. HbA1c < 10% did not compromise implant survival, but worsened PD and MBL were seen with increasing HbA1c levels in a dose-response manner.
de-Oliveira-Neto et al., 2019 [11]	Overview of Systematic Reviews	Eight systematic reviews (6 moderate-quality, 2 high-quality based on AMSTAR)	Dental implant placement in diabetic patients	Not specified (general population focus)	Implant survival rate, marginal bone loss	Implant survival: 31.8%–100%; Diabetes effect on survival: None (4 meta-analyses); Marginal bone loss: Statistically significant but clinically irrelevant	Diabetes does not significantly affect implant survival rates. Marginal bone loss was statistically affected, but not to a clinically relevant extent. Recommendations from studies with moderate methodological quality should be applied cautiously.

Quality Assessment

The quality assessment of the included studies, detailed in Table 2, was conducted using the AMSTAR 2 tool, with additional metrics applied where appropriate. Two studies were rated as high quality due to their robust methodological frameworks, including comprehensive literature searches, rigorous risk-of-bias assessments, and adherence to reporting standards like PRISMA and PROSPERO registration. Another study achieved a moderate-quality rating due to challenges such as high heterogeneity (I² > 70%) and limited sample sizes, despite strengths in biomarker assessment and dose-response meta-analysis. The final study, an overview of systematic reviews, was rated moderate to high quality, reflecting variability in the methodological quality of the included reviews. These assessments underscore the credibility of the findings while highlighting areas for caution in interpreting results with greater heterogeneity or methodological limitations.

**Table 2:** Quality assessment of the included studies. AMSTAR 2: A Measurement Tool to Assess Systematic Reviews 2; DRMA: Dose-Response Meta-Analysis; REMR: Robust Error Meta-Regression; I²: Measure of Statistical Heterogeneity in Meta-Analyses; PRISMA: Preferred Reporting Items for Systematic Reviews and Meta-Analyses; PROSPERO: International Prospective Register of Systematic Reviews

Author(s) and Year	Tool Used	Key Domains Assessed	Quality Score/Rating	Overall Assessment
Schimmel et al., 2018 [8]	AMSTAR 2	- Clear research question and inclusion criteria defined - Comprehensive literature search - Risk of bias assessment - Adequacy of meta-analytic methods - Heterogeneity management	High Quality	High-quality systematic review with well-defined criteria, comprehensive search, and robust meta-analytic methods. Potential limitations include limited subgroup analyses for systemic conditions.
Andrade et al., 2022 [9]	AMSTAR 2	- PROSPERO registration - PRISMA adherence - Rigorous bias analysis (Joanna Briggs Institute tool) - Consistent use of statistical methods - Addressed heterogeneity with I² and subgroup analyses	High Quality	Methodologically strong review with rigorous bias assessment, PROSPERO registration, and adherence to PRISMA. Comprehensive analysis of glycemic control impact, though more data on long-term outcomes could enhance the review.
Lv et al., 2022 [10]	AMSTAR 2 + DRMA Robust Error Meta-Regression (REMR)	- Comprehensive database search - Dose-response meta-analysis - Sensitivity analysis - Thorough biomarker assessment - Clear reporting of statistical heterogeneity	Moderate Quality	Comprehensive in biomarkers and dose-response analyses. Moderate quality due to high heterogeneity (I² > 70% in multiple analyses) and a smaller number of included studies.
de Oliveira-Neto et al., 2019 [11]	AMSTAR 2	- Clear research question - Quality assessment using AMSTAR - Descriptive synthesis of findings - Inadequate reporting of heterogeneity - Moderate methodological quality in many included reviews	Moderate to High Quality	Solid methodological evaluation of systematic reviews. Some included reviews were of moderate quality, and findings on marginal bone loss require cautious interpretation. Recommendations for clinical practice are appropriately cautious.

Discussion

The systematic review underscores the robust survival rates of dental implants in both diabetic and non-diabetic populations, reinforcing their viability as a reliable treatment option across diverse groups. Schimmel et al. [8] reported particularly high survival rates in geriatric patients and individuals with systemic conditions, including T2DM, with a 1-year survival rate of 97.3% (95% CI: 94.3-98.7) and a 5-year survival rate of 96.1% (95% CI: 87.3-98.9). These findings highlight the resilience of dental implants even in patients with complex systemic conditions, provided that appropriate management protocols are followed. This evidence aligns with the broader understanding that diabetes, when well-controlled, does not pose a significant barrier to achieving favorable outcomes in dental implantology.

Similarly, Andrade et al. [9] corroborated these findings by demonstrating no statistically significant differences in implant survival rates between type 2 diabetic patients and their non-diabetic counterparts (RR = 1.00, 95% CI: 0.96-1.04, p = 0.91). Furthermore, their analysis revealed that marginal bone loss between immediately loaded implants and conventionally loaded implants in diabetic patients was negligible (MD = -0.08, 95% CI: -0.25-0.08, p = 0.33). These outcomes underscore the critical role of strict glycemic control and adherence to clinical protocols in mitigating potential complications. Collectively, these findings emphasize that diabetes, particularly T2DM, need not be a contraindication for dental implants if managed appropriately, highlighting the importance of individualized patient care and meticulous planning to optimize outcomes.

Lv et al. [10] added depth to these findings by focusing on peri-implant biomarkers and clinical outcomes in hyperglycemic patients. They identified significantly higher levels of negative regulators of bone metabolism, such as IL-6, TNF-α, and RANKL (p = 0.01), in diabetic patients compared to healthy individuals, alongside worsened peri-implant outcomes like probing depth (PD) and marginal bone loss (MBL) (p < 0.01). Interestingly, implant survival was not compromised when HbA1c levels were below 10%, though dose-response analyses revealed progressively worse outcomes with higher HbA1c levels. Oliveira-Neto et al. [11] supported the notion that diabetes does not significantly affect implant survival rates, with survival ranging from 31.8% to 100% across systematic reviews. However, they noted that MBL was statistically significant but clinically irrelevant, indicating that caution is needed when interpreting findings from moderate-quality studies. Collectively, these studies emphasize that while diabetes and hyperglycemia pose risks to peri-implant health, implant survival remains favorable with proper management and glycemic control.

The findings of this systematic review are consistent with a substantial body of existing literature, which indicates that type 2 diabetes mellitus, when adequately controlled, does not significantly affect dental implant survival rates [12]. Numerous studies have demonstrated this conclusion, including meta-analyses conducted by Chrcanovic et al. [13] and Monje et al. [14], which found comparable survival rates between diabetic and non-diabetic populations under well-managed glycemic conditions. This evidence underscores the resilience of dental implants in diabetic individuals, provided that strict glycemic management is maintained. Notably, studies such as those by Schimmel et al. [8] and Andrade et al. [9] further strengthen this understanding by showing high implant survival rates in diabetic patients who adhered to clinical protocols. These findings highlight the critical importance of multidisciplinary care and adherence to best practices to minimize the risk of complications and ensure optimal implant outcomes.

Moreover, the review’s findings regarding MBL align closely with prior research, such as the work of Lv et al. [10]. While diabetes, particularly in poorly controlled cases, is associated with elevated inflammatory markers like IL-6 and TNF-α, the impact on implant survival appears to be minimal when glycemic levels are well-regulated. Although increased levels of these inflammatory markers may adversely influence peri-implant health, the clinical significance of these effects remains limited in terms of long-term implant success [15]. These insights emphasize the nuanced relationship between systemic inflammation and dental implant outcomes, suggesting that while diabetes may introduce specific challenges, careful patient management and monitoring of glycemic levels can effectively mitigate risks. The overall findings advocate for a proactive, evidence-based approach to managing diabetic patients undergoing implant procedures.

However, certain discrepancies in the literature warrant further exploration to address lingering uncertainties about the impact of diabetes on dental implant success. de Oliveira-Neto et al. [11] highlighted significant variations in MBL reported across systematic reviews, suggesting that these differences may be attributable to inconsistencies in study populations, varying follow-up durations, or the methodologies employed to evaluate peri-implant outcomes. These disparities underscore the need for standardization in research protocols to enhance the comparability of findings. Furthermore, studies have indicated that poorly controlled diabetes, particularly with HbA1c levels exceeding 8%, may exacerbate peri-implant complications such as increased MBL and inflammatory responses [16,17]. These findings are echoed by Lv et al. [10], who identified a dose-response relationship between HbA1c levels and peri-implant health metrics, with worsening outcomes observed as glycemic levels rise. This evidence reinforces the notion that precise glycemic control is critical for minimizing complications and optimizing implant success in diabetic patients.

This systematic review contributes significantly to the field by integrating these nuanced insights and emphasizing the importance of glycemic thresholds and peri-implant biomarkers in understanding implant success in diabetic populations [18]. By synthesizing data from multiple systematic reviews, it establishes a robust framework for guiding clinical decision-making. The findings highlight the resilience of dental implants even in diabetic individuals, provided glycemic levels are adequately managed, and emphasize the need for ongoing metabolic regulation and interdisciplinary care. This comprehensive approach enables clinicians to navigate the complexities of diabetes management in implant dentistry while ensuring optimal outcomes. Additionally, the review paves the way for future research aimed at exploring advanced glycemic thresholds, peri-implant health metrics, and the long-term implications of sustained hyperglycemia on implant survival and peri-implant complications. Such endeavors will be crucial for addressing the gaps in current knowledge and developing evidence-based strategies for managing diabetic patients undergoing implant procedures.

The findings of this systematic review hold significant clinical implications, emphasizing that dental implants can be a viable and reliable treatment option for patients with type 2 diabetes mellitus, provided glycemic control is adequately managed [19]. Clinicians should prioritize preoperative and postoperative glycemic monitoring, aiming to maintain HbA1c levels below 8% to minimize peri-implant complications such as MBL and inflammatory responses [20]. Strict adherence to established surgical protocols, including proper patient selection, meticulous surgical techniques, and effective maintenance of oral hygiene, is critical to optimizing outcomes [21]. Furthermore, incorporating personalized treatment plans that consider the patient’s systemic health and inflammatory status, as highlighted by biomarkers like IL-6 and TNF-α, can further enhance success rates. These recommendations underline the importance of interdisciplinary care involving endocrinologists, dentists, and hygienists to ensure comprehensive management and long-term implant stability in diabetic patients.

This systematic review demonstrates significant strengths, including a comprehensive literature search across multiple databases, ensuring a wide scope of relevant studies was captured. The rigorous application of quality assessment tools such as AMSTAR 2 [22] provided a robust evaluation of the methodological quality of included reviews, enhancing the credibility of the findings. Additionally, advanced statistical techniques, such as the dose-response meta-analysis in Lv et al. [10], offered nuanced insights into the relationship between glycemic control and peri-implant outcomes, contributing to a deeper understanding of the subject. However, the review is not without limitations. The quality and heterogeneity of the included studies, with varying methodologies and follow-up durations, present challenges in drawing uniform conclusions. Some studies had limited sample sizes, reducing the generalizability of findings, particularly to populations with poorly controlled diabetes or comorbidities. Furthermore, the potential for publication bias and the lack of data from underrepresented regions may have influenced the breadth of evidence [23]. These limitations highlight the need for caution when interpreting results and underscore the importance of future research with standardized methodologies and broader population inclusion.

This review identifies several gaps in the current literature that warrant further investigation. Long-term studies examining MBL and implant survival in diabetic patients, particularly those with poorly controlled glycemic levels, are needed to better understand the impact of sustained hyperglycemia on implant outcomes [24]. Subgroup analyses focusing on specific glycemic thresholds (e.g., HbA1c <7%, 7-8%, >8%) and their influence on peri-implant health would provide clearer guidance for clinical decision-making. Additionally, the lack of standardized reporting across studies, particularly in defining peri-implant outcomes and follow-up durations, limits the comparability of findings. Clear, practical guidelines for clinicians are essential, including recommendations to aim for HbA1c levels below 7% whenever possible to optimize implant survival and peri-implant health. Future research should adopt consistent protocols and outcome measures to enhance the reliability of meta-analyses and facilitate the development of evidence-based recommendations for implant placement in diabetic populations. Addressing these gaps would significantly advance the understanding and management of dental implants in this growing patient group.

## Conclusions

This systematic review highlights that dental implants are a viable and reliable treatment option for patients with type 2 diabetes mellitus, provided glycemic control is adequately maintained. Implant survival rates were consistently high, with no significant differences observed between diabetic and non-diabetic populations when HbA1c levels were well-managed. However, MBL and peri-implant health metrics were adversely affected in cases of poor glycemic control, underscoring the importance of metabolic regulation and strict adherence to clinical protocols. While the findings reaffirm the feasibility of dental implants in diabetic patients, they also emphasize the need for personalized treatment strategies and interdisciplinary collaboration to optimize outcomes. The review identifies key research gaps, such as the need for long-term studies and standardized outcome reporting, providing a foundation for future investigations to enhance evidence-based practice.
